# Food procurement in upper secondary schools in a Norwegian county: nutritional quality and environmental impacts

**DOI:** 10.29219/fnr.v69.12600

**Published:** 2025-11-11

**Authors:** Marie M. Bjøntegaard, Mari Mohn Paulsen, Bob van Oort, Lene Frost Andersen

**Affiliations:** 1 Department of Nutrition, Institute of Basic Medical Sciences, Faculty of Medicine, University of Oslo, Oslo, Norway; 2 Department of Food Safety, Norwegian Institute of Public Health, Oslo, Norway; 3 Centre for Sustainable Diets, Norwegian Institute of Public Health, Oslo, Norway; 4 CICERO, Centre for International Climate Research, Oslo, Norway

**Keywords:** Food procurement, nutritional quality, life cycle assessment, upper secondary schools, school meal

## Abstract

**Background:**

Public food procurement has the potential to play a significant role in transforming the food system.

**Objective:**

This study aimed to investigate the nutritional quality and environmental impacts of food procurement in public upper secondary schools within a large county in Norway.

**Design:**

A cross-sectional study with food procurement data from 35 upper secondary school canteens, analysed using a food-and-nutrient calculation system at the University of Oslo, which also includes a life cycle assessment (LCA) food database.

**Results:**

Food procurement amongst school canteens did not align with guidelines for food and meals in upper secondary schools and recommendations for nutritional considerations in public food procurement. There was considerable variability between the schools’ food procurement regarding nutritional quality and environmental impacts. However, on average, high levels of saturated fat and added sugar, as well as inadequate levels of folate, vitamin D, iron and iodine, were observed. Red meat and dairy products exhibited the most significant environmental impacts between the food groups.

**Discussion:**

Few studies have utilised food procurement data to evaluate nutritional quality and environmental impacts of school meals. Using food procurement instead of actual consumption data introduces some uncertainties, including limited knowledge about the amount of food waste, quantities actually consumed and demographics of the canteen users. Identifying key nutrients of concern can be invaluable in guiding meal planning and food procurement, especially in a school setting. Our environmental analysis supported current literature by illustrating the high impact of animal-based foods relative to plant-based foods.

**Conclusions:**

The present study found both nutritional and environmental limitations in food procurement in public upper secondary schools in a large Norwegian county. Encouraging procurement of plant-based proteins and sustainably sourced fish whilst reducing purchases of full-fat dairy products would better align with the guidelines for food and meals in schools and reduce the environmental impact. Moreover, significant variability in procurement practices that do not comply with the guidelines suggests a need for clearer guidance and follow-up.

## Popular scientific summary

- Public food procurement may enable food system transformation and ensure adequate nutrition across population groups.
- The nutritional quality and environmental impact of purchases from 35 public upper secondary school canteens in a large Norwegian county were examined.
- Food procurement amongst school canteens did not comply with guidelines. Several opportunities for improvement have been identified.
- Strategic tools and monitoring may be beneficial for ensuring healthier and more sustainable food procurement.

Public food procurement, which involves the purchase of foods and beverages by public institutions, has recently gained momentum for its potential use as a policy instrument for enhancing both public and planetary health, illustrated by several large European Union (EU)-funded projects (1–3). Public food procurement can promote healthy foods from sustainable food systems, ensuring food security and protecting environmental, sociocultural and economic components of sustainability (4). Additionally, food provided in the public sector can reach a broad range of individuals, including potentially vulnerable groups like schoolchildren, patients in hospitals or elderly residents in care homes (5).

A monitoring initiative focused on food systems transformation in the countdown to the 2030 sustainability goals has highlighted the critical role of governance in driving transformative change (6). Public food procurement may act as a valuable mechanism for enabling this transformation. In Norway, public meals are provided in institutions such as kindergartens, schools, hospitals, military services and more, resulting in a large number of meals being served daily. To ensure high nutritional quality of these meals, the Norwegian Directorate of Health has issued guidelines for nutritional considerations in public food procurement (7) (Supplementary Table 1). These guidelines outline food group recommendations, focusing on meal planning and the purchase of specific products. Furthermore, the Norwegian Agency for Public and Financial Management (DFØ) has developed criteria for public food procurement, focusing on food waste, climate-friendly and organic foods, deforestation, packaging and human rights (8).

Schools represent a particularly effective arena for public health initiatives, given their wide reach and their potential role in facilitating healthy habits that can support children and adolescents as they transition into adulthood. Enhancing the procurement of healthy and sustainable foods in schools can therefore serve as a multi-purpose strategy, by reducing the risk of malnutrition and the development of non-communicable diseases whilst also promoting sustainable food choices (5).

In Norway, adolescents aged 16–24 years are entitled to 3 years of upper secondary school upon completing primary and lower secondary school. The Norwegian government does not currently provide free school meals; however, guidelines exist for meal planning and product assortment available for purchase in upper secondary schools (9) (Supplementary Table 2). The guidelines emphasise that school canteens should promote students’ well-being and health by having a food offering guided by the Norwegian food-based dietary guidelines (9). Most students bring home-packed lunches that typically consist of a bread-based meal. However, as students age, they tend to purchase foods from the school canteen or nearby shops (10). The organisation of school canteens and their food offerings vary from school to school, but they generally provide simple meals such as sandwiches, salads, soups, yogurt, beverages and fruit, with some canteens also offering breakfast options.

Several studies have investigated adherence of school meals to guidelines. Most of these have focused on school lunch menus (11–15) or student purchasing behaviour (16, 17), whereas few studies have used procurement data for the evaluation of schools meals (18, 19). There have been few investigations into the nutritional quality and environmental impact of public food procurement in Norway. Van Oort, Holmelin and Milford (20) estimated that public food procurement in Norwegian counties and municipalities contributed to 4% of the total national greenhouse gas emissions in 2019, based on a national upscaling of greenhouse gas emissions from public food procurement in Oslo municipality. Their investigation of purchases by two upper secondary schools revealed that emissions were driven by meat, dairy and beverages, and that they purchased more meat, sugar and composite meals, and less eggs, dairy products, fruits and berries compared to nursing homes and for Oslo municipality as a whole. Nutritional quality of food procurement has been investigated in 10 upper secondary schools in the same county as the present study using Nutri-Score for ranking (18). The Nutri-Score is a front-of-pack label that rates the nutritional quality of food products from A (healthiest) to E (least healthy). The study found varying nutritional quality of the foods offered in these schools.

To address existing data gaps in food procurement practices, the aim of the present study was to investigate the nutritional quality and environmental impacts of food procurements in public upper secondary schools in a large Norwegian county.

## Methods

### Study design and sample

In this cross-sectional study, electronic invoice food procurement data from upper secondary schools were linked to an in-house food-and-nutrient calculation system (KBS, database AE 22) at the University of Oslo (21), which further includes a life cycle assessment (LCA) food database (22).

The main sample consisted of 33 public upper secondary schools located in a large Norwegian county in 2022, with 35 independently organised school canteens operating in different locations. Schools in the county were included in the analysis if their canteens were managed directly by the school. Canteens were excluded if the management of the canteen was outsourced to an external provider, if no canteen was operating during the specific study period or if no invoice data were available. Private schools were also excluded.

### Procurement data

The procurement department of the studied county maintains an overview of purchases made by all public institutions within the county by collecting invoices in electronic trading format from the suppliers. Procurement data from the 35 school canteens included in the study were obtained in Microsoft Excel 365 and comprised purchases made for the school canteens during either the first or second quarter of 2022. The dataset also included purchases made outside of established procurement contracts, for example, purchases from local stores. Procurements for vending machines and other available food options that were not managed by the schools’ canteens were not included, and the extent to which these options are available in the schools is unknown. The dataset included information about the product ID, supplier, product name, unit type, number of units, price, time period and school canteen.

### Data processing

The electronic invoice information was recorded differently depending on the supplier, leading to varying quality of the recorded data. To address this, extensive data processing was required. This included a data clean, exclusion of non-food items from the dataset and identification and standardisation of units and estimation of volumes in grams. The steps involved in identification and standardising units in the procurement data are described as follows.

Step 1 involved the identification of units and product volume. As previously mentioned, the dataset did not explicitly state the unit and purchased volume. Instead, available information such as product name, unit type (carton, each, etc.), quantity and cost was used to estimate volume. This step was conducted in two ways due to the varying quality of the invoice data: Step 1a involved procurements *with* a description of the volume and unit in the product name (~80%). Information about the unit and product weight was extracted from the product title, that is, ‘Feta cheese 1KG’, and transferred into a separate column in Microsoft Excel. Due to the different formats from various suppliers, we utilised a combination of different methods, including formulas, colour coding, and the search and filter functions. Alternatively, in step 1b, for procurements lacking a volume description in the product name (~20%), volume was estimated by comparing the price per unit to similar products. If no similar products with known volumes were available, we applied generic size-estimates from the Norwegian report ‘Weights, measures and portion sizes for foods’ (23) or the median size-estimation of foods available in the food composition database KBS (21).

In step 2, we calculated the total procured volume by multiplying the product volume with purchased quantity. Furthermore, all units were standardised to grams with the aid of conversion factors available from the report ‘Weights, measures and portion sizes for foods’ (23), the in-house food-and-nutrient calculation system KBS (21) and units in gram for various single-serve ice creams from Matinfo.no.

In step 3, under- and overestimation of volumes were identified by comparing the price per kilogram within product groups to detect anomalies by evaluating similar products.

### Assessment of nutritional quality

The KBS database includes dietary information of more than 4,000 food items available in Norway; however, the level of details varies from brand-specific to more generic food items. To assess the nutritional quality of the purchases, we selected the closest corresponding food item from the KBS database. This process utilised brand-level information and the level of processing or preparation indicated in product names in the procurement lists, when available, to match these with the corresponding items and preparation level in KBS. Food items may distinguish between origin, preparation and processing level (including fortification). For food items with unknown origin, preparation or processing level in the procurement data or the KBS database, generic values were used as a proxy.

The food grouping utilised in the present study was based on the food grouping from a national dietary survey (24). Additionally, to better incorporate a focus on environmental sustainability, elements from the food grouping used by Lengle et al. (25) were applied. Furthermore, due to the high number of purchases of various beverages and dairy products, extra focus was placed on these products. Composite dishes were split into the corresponding ingredients.

The micronutrients and fibre contents in the food procurement were standardised to per 10 MJ, to adjust for school sizes and to allow for comparison with recommendations for dietary planning for groups (26). Macronutrient content was presented as the percentage of total energy originating from either protein, carbohydrates or fats (E%). We selected a limited range of nutrients for analysis, specifically those relevant to the age group, due to the uncertainty associated with purchase data and the estimation of volumes. The nutrient content of all food and beverages was calculated per edible share and accounted for weight changes for pre-heated foods if applicable, according to Dalane et al. (23).

### Estimation of environmental impacts

The environmental impacts of the procurements were estimated using a recently incorporated Norwegian LCA food database as part of the KBS infrastructure (22). The environmental impacts evaluated in the present study include global warming potential with a 100-year time horizon (kg CO_2_-eq), freshwater eutrophication (kg P-eq), acidification of soils (kg SO_2_-eq) and land use (m2a). In short, the database is based on published LCA data from scientific literature and LCA databases, covering impacts from farm to fork. The database was made representative of foods in Norway by using Norwegian LCA data if available, investigating import statistics and adapting distribution to Norway for imported foods. Food waste was not systemically assessed in the database; however, it was included if analysed in the original LCA data.

### Statistical analysis

Descriptive statistics were used to describe the schools’ food procurement in terms of distribution across food groups, nutrient content and environmental impacts. To investigate the variation between schools and guide the presentation of data, we performed normality tests using visual inspection of histograms and the Shapiro-Wilks test from SPSS statistics (version 30).

To describe the contribution in grams of various main food groups and specific foods and beverages (referred to as subgroups) to the schools’ food procurement, the median, 25th percentile (Q1) and 75th percentile (Q3) were calculated, due to the non-normal distribution of the data. Additionally, the mean and standard deviations (SDs) were also presented. To describe the contribution of main food groups and subgroups, we utilised median values. The formula used to calculate the percentage shares of main food group and subgroups, where food groups are denoted as FG and the subscripts represent individual food groups, is as follows:

$$\%\text{-share of }{\text{FG}}_{1}=({\text{FG}}_{1}*100)/({\text{FG}}_{1}+\ldots +{\text{FG}}_{13})$$

All nutrients were displayed as mean and SDs. For environmental impacts, the median contribution of food procurement was presented, as per 10 MJ, along with Q1 and Q3. Finally, we created radar plots in Excel to illustrate the contributions of various food groups to the environmental impacts.

## Results

### Characteristics of the sample

The sample consisted of 33 upper secondary schools with 35 independently organised school canteens at different locations within a large Norwegian county (Table 1). Four school canteens were excluded due to a lack of invoice information, and one school was excluded due to uncertainty about whether the canteen was privately or publicly managed. During the 2022 school year, the majority of these schools had between 500 and 1,000 students. The upper secondary schools were primarily classified as urban schools according to the classification of urban settlements by Statistics Norway (27).

**Table 1:** Characteristics of the upper secondary school canteens (*n* = 35)

	Student enrolment					
	<500^a^		500-1000		>1000	
	n	%	n	%	n	%
School canteens^b^	12	34	17	49	6	17
Urban location^c^	9	75	17	100	6	100
Rural location^c^	3	25	0	0	0	0

a Student enrolment for each campus was based on the school year of 2022, as recorded by the Norwegian Directorate for Education and Training (42). The school was contacted directly if student number was not found.

b The school canteens were located at different campuses in various locations.

c The school canteen locations were classified as either urban settlements or not, by using the definition and map by Statistics Norway (27).

### Procurements of foods and beverage items

**Fig. 1:**
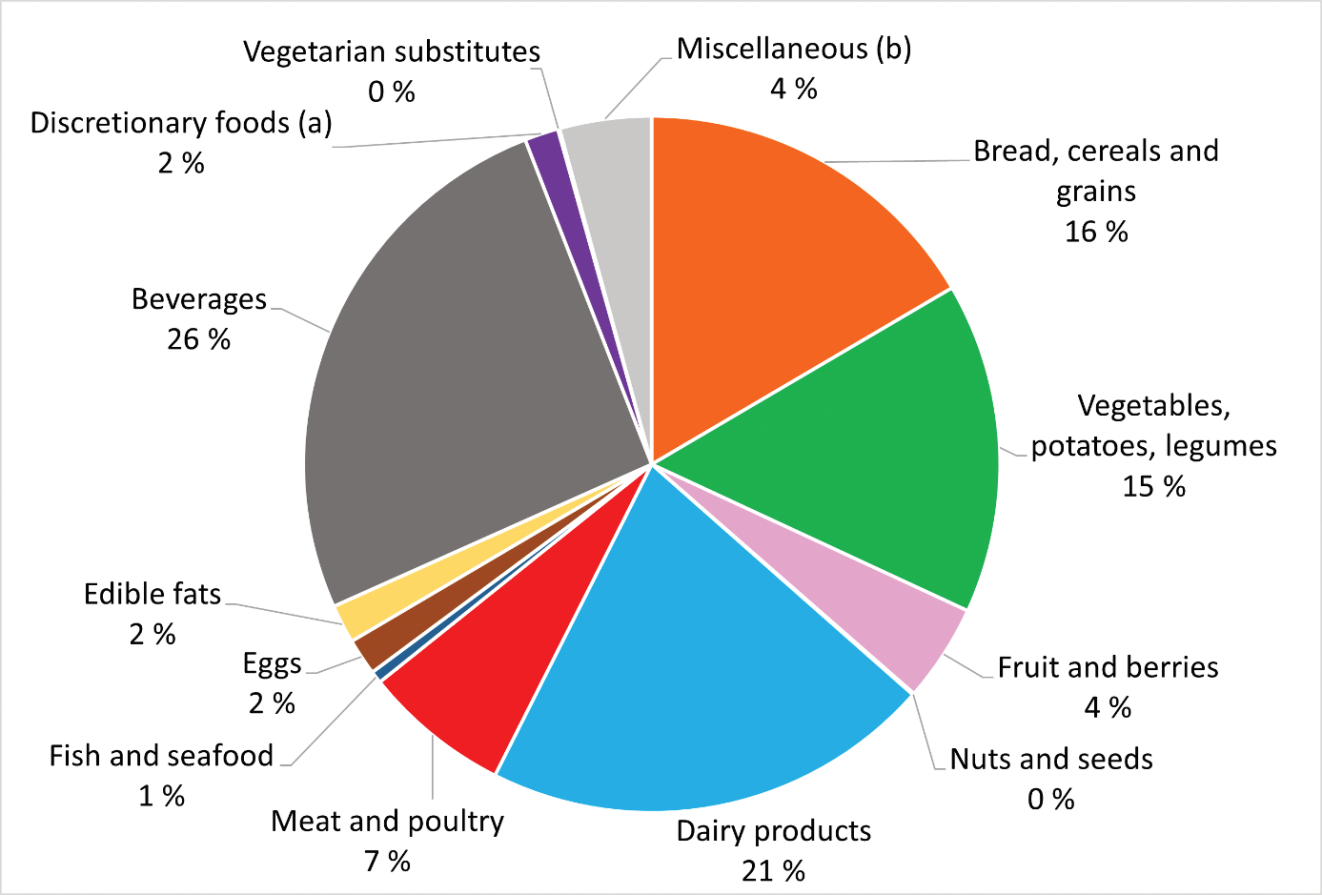
Percentage contribution (based on median amount in grams) of food groups, excluding inedible parts, to the total procurements amongst 35 upper secondary school canteens. ^a^Discretionary foods include chips, popcorn, other snacks, cakes, cookies and sweet pastries, chocolates and other sweets, ice lollies and sorbet, and sweet spreads. Dairy-based ice cream is included as a dairy product. ^b^Miscellaneous items include sauces and condiments, spices, dressing, sugar, honey and sirup, and powders.

Figure 1 illustrates the food groups purchased by the school canteens. Due to skewed data, we have focused on the median contributions; however, the purchases varied between the school canteens, as further described in Supplementary Table 3. *Beverages* represented the largest category, accounting for a median contribution of 26% of total purchases by weight. The variation in purchases between school canteens was especially large for beverages, in which 50% of schools procured between 498 and 1,492 litres during the quarter. Within this category, soft drinks were most purchased, comprising 40% of procurements. Sugar-sweetened iced tea accounted for 73% of these purchases, whereas artificially sweetened soft drinks contributed with the rest. Bottled water accounted for 30% of procurements. *Dairy products* were the second largest food group procured, constituting 21% of the total purchases by weight. Within this group, milk contributed with 62% of purchases, including 92% low-fat milk (1% fat), whilst full-fat cheese contributed with 18%. *Bread, cereals and grains* made up 16% of the total purchases by weight, with bread alone accounting for half of this category. Of the bread purchases, the majority were white (67%) or coarse bread (30%). Despite the considerable variation in the purchase of white and coarse bread across school canteens, there were, in general, few purchases of extra course bread. *Vegetables, legumes* and *potatoes* contributed with 15% of the total amount of purchases by weight, whereas fruits and berries had a relatively low share of 5%. Large variations between school purchases were found for both vegetables (291–603 kg), potatoes (35–148 kg) and fresh or frozen fruit (88–258 kg). Regarding meat (7%), half of the purchased amount of *meat* was red meat, with a large share being processed products (62%). In contrast, for poultry, purchases were primarily whole cuts or minced (88%). *Fish* and *seafood* accounted for a very small share of purchases (1%). Most of the *edible fats* comprised of plant-based fats (77%).

When comparing the school canteens purchases of food groups to the guidelines for food and meals in upper secondary schools (9) and nutritional considerations for public food procurement (7) (Supplementary Tables 1 and 2), we observed some discrepancies. There was a relatively high procurement of white bread, full-fat cheese, highly processed red meat, sugar-sweetened yoghurt and soft drinks, whilst the procurement of fruit and berries, fish and seafood, and nuts and seeds were low. In line with the guidelines, school canteens procured mostly low-fat (1% fat) milk, and most of the white meat consisted of less-processed meat. The school canteens also purchased more plant-based fats than animal-based. Vegetable oils and table margarine were common purchases in line with the guidelines for edible fats; however, hard margarine and particularly dairy-based butter were also regularly purchased amongst some school canteens (data not shown). The guidelines recommend using a varied selection bread spreads that includes meat, fish and vegetables (9). However, due to the low contribution of fish, eggs, legumes or meat analogues amongst procurements, it may be assumed that the selection of spread for sandwiches was not varied.

### Nutritional assessment of procurements

Table 2 presents the average nutrient content in the purchased food items, standardised per 10 MJ and compared against the Norwegian recommendations for dietary planning for groups (26).

**Table 2:** Mean nutrient content of food procurements from 35 upper secondary school canteens, presented per 10 MJ and according to the Norwegian nutrient recommendations for dietary planning for groups (26)

Nutrients	Procurement data/10 MJMean (SD)	Recommendations for dietary planning for groups^a^
Protein, E%	16 (2)	15
Fats total, E%	37 (5)	32–33
Saturated fat, E%	13 (3)	<10
Carbohydrates total, E%	44 (5)	52–53
Free sugar, E%	9 (3)	<10
Added sugar, E%	8 (3)	
Dietary fibre (g)	32 (9)	30
Vitamin D (μg)	4 (2)	14
Folate (μg)	280 (49)	450
Vitamin B12 (μg)	5 (1)	2
Calcium (mg)	1,102 (230)	1,000
Iron (mg)	11 (2)	16
Iodine (μg)	123 (33)	170

a Based on Norwegian Directorate of Health (26) and presented as nutrient per 10 MJ.

For macronutrients, the percentage of energy (E%) from protein, total fat and saturated fat was found to be 16 E%, 37 E% and 13 E%, respectively, all of which were above the recommended levels. The percentage of energy from carbohydrates was lower than recommended (44 E%), with a relatively high proportion of energy coming from added sugar (8 E%), although this was still within the recommended daily range. Furthermore, the purchased food items contained a sufficient level of dietary fibre.

Regarding micronutrients, the contributions from the food procurements were within recommended levels for vitamin B12 (5 μg) and calcium (1,102 mg). However, the procured level of folate (280 μg), vitamin D (4 μg) and iron (11 mg) was below the target values for dietary planning for groups, even with mean + 2 SDs. A low level of iodine was also seen (123 μg), which only reached the target value (170 μg) with mean + 2 SDs.

### Environmental impact of the food procurements

**Fig. 2:**
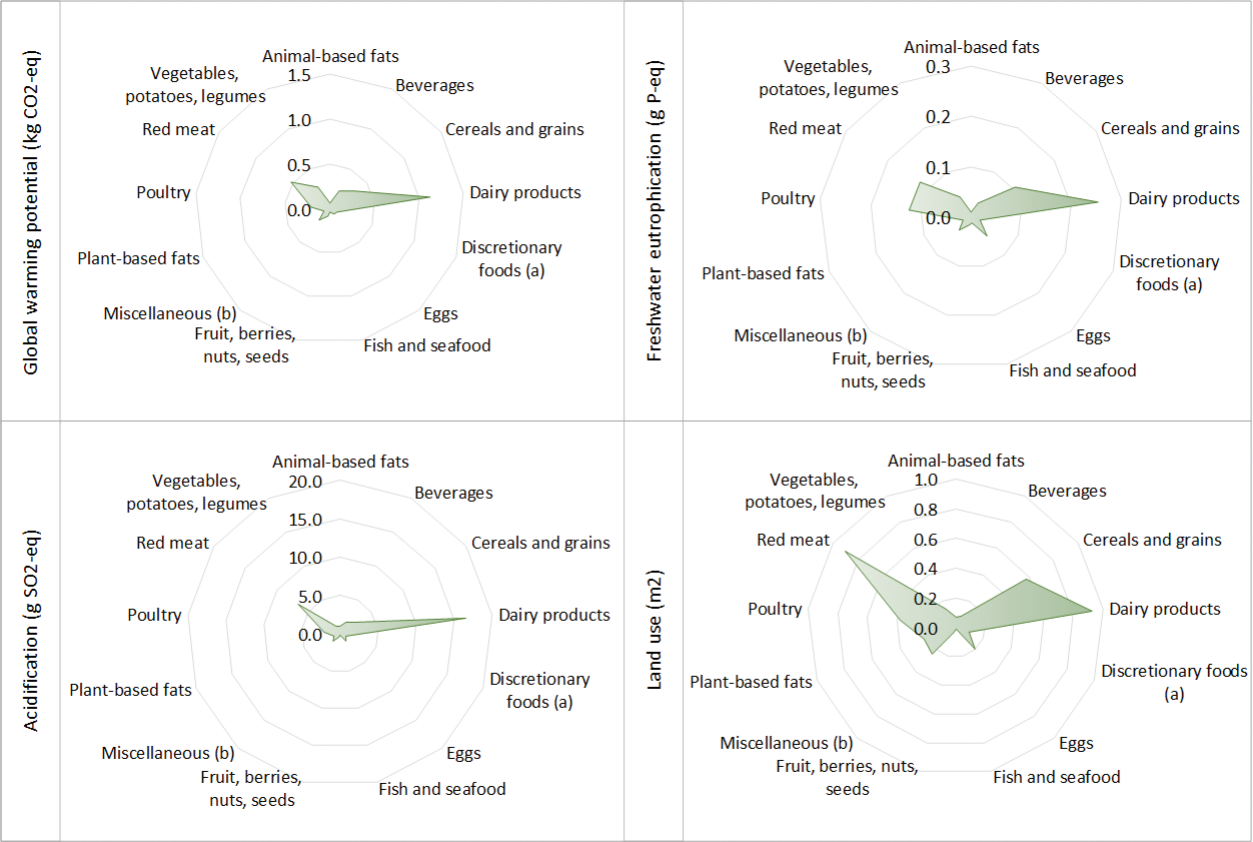
Food group contribution to environmental impacts per 10 MJ. ^a^Discretionary foods include chips, popcorn, other snacks, cakes, cookies and sweet pastries, chocolates and other sweets, ice lollies and sorbet, and sweet spreads. Dairy-based ice cream is included as a dairy product. ^b^Miscellaneous items include sauces and condiments, spices, dressing, sugar, honey and sirup, and powders.

Table 3 shows the environmental impact associated with the food procurements per 10 MJ. Furthermore, Fig. 2 presents radar plots displaying the relative contribution of food groups to the four categories of environmental impact.

**Table 3:** Median (Q1, Q3) environmental impacts of food procurement in 35 upper secondary school canteens, presented per 10 MJ

Environmental impacts	Unit	Median	Q1^a^	Q3^b^
Global warming potential, 100y	kg CO2-eq/10 MJ	3.6	3.3	4.0
Freshwater eutrophication	g P-eq/10 MJ	0.9	0.9	1.0
Acidification	g SO2-eq/10 MJ	41.4	34.9	45.2
Land use	m^2^/10 MJ	4.1	3.8	4.5

a Q1 = 25^th^ percentile.

b Q3 = 75^th^ percentile.

For global warming potential (100 year), the median contribution from the schools’ food procurement was 3.6 kg CO_2_-equivalents (eq). Dairy products were the largest contributor to global warming potential, followed by red meat and cereals and grains. For freshwater eutrophication, the median contribution was 0.9 g P-eq., and the main contributing food groups were dairy products and meat, followed by cereals and grains. The level of soil acidification was 41.4 g SO2-eq. Acidification was mainly driven by dairy products and red meat. Land use throughout the life cycle of the procured foods was estimated to be 4.1 m^2^, mainly driven by dairy products and red meat, followed by cereals and grains and poultry.

## Discussion

The present study describes the nutritional quality and the environmental impact of food procurement in public schools within a large Norwegian county. We identified several opportunities to improve procurement to be more nutritious whilst reducing the environmental impact of purchases.

Results from the present study indicated that, although there was some variability between schools, the food procurement in many upper secondary schools did not comply with the recommendations for public food procurement (7) and guidelines for food and meals in upper secondary schools (9). This was due to the high purchase levels of white bread, full-fat cheese, highly processed red meat, sugar-sweetened yoghurt and soft drinks, along with low purchase levels of fruit and berries, fish and seafood, and nuts and seeds. Moreover, our findings suggest a lack of variety in the spreads used for sandwiches and other lunch meals, where the guidelines recommend including spreads based on plant-based products as well as fish and seafood (9).

Only a few studies have utilised procurement data to evaluate schools meals (18, 19). Although there are several investigations of the nutritional quality and/or environmental impact of school meals in the literature, other approaches have more frequently examined lunch menus (11–15), observed student purchases (16) or conducted questionnaires amongst canteen staff (28). These studies, based on primary, lower and upper secondary schools in several high-income countries, demonstrate that school meals often do not align with national guidelines (11, 13, 28), and that improvements can be made to improve both the nutritional quality and environmental impact (12, 14–16). Despite the different methodologies used for analysis, our results based on food procurement data support findings in the above-mentioned studies, thereby suggesting that analysis of food procurement may be a useful method for these evaluations.

In a Danish study on compliance with food service guidelines for hot meals, upper secondary schools were found to score low, with only 10% of schools (*n* = 86) complying with all five nutrition criteria, including servings of fruit and vegetables, fish, high-fat dairy, and meat and refined grains (28). The authors observed higher compliance with food service guidelines when there was a high share of organic procurement (>50%) versus a low share (<50%).

Surprisingly, the procurement of soft drinks was high across schools in the present study, despite the recommendation not to have these beverages available for sale in upper secondary schools. Our finding on soft drink consumption is supported by a secondary analysis of data from a dietary survey amongst Norwegian children and adolescents, which found that 78% of 8th graders (12–14 years) regularly consumed soft drinks (29). The immediate availability of foods at school can significantly influence food choices (30), suggesting that procurement practices in upper secondary schools may play a crucial role in the intake of these beverages.

In our assessment of the nutritional quality of food procurements, we compared the nutrient content from food procurement against the recommendations for dietary planning for groups, as these guidelines are intended for meal planning in the educational and health sectors (26). However, the comparison between public food procurements in the schools and the recommendations for dietary planning for groups (26) has certain limitations. The recommendations are based on nutrient requirements for individuals aged 6–65 years, for both sexes, and consider actual food consumption over a full day. In contrast, our data reflect *purchases*, not consumed amounts, presumably intended for lunch, breakfast or a snack.

We observed a high proportion of total fat and saturated fat in the food procurements. Since most of the cheese purchased was full-fat, opting for low-fat cheese and incorporating more plant-based and fish-based spreads for bread, as recommended by the Norwegian Directorate of Health (9), could reduce the levels of total fat and saturated fat in the procurements. In terms of micronutrients, the levels of folate, vitamin D and iron were below the target values for dietary planning for groups with mean + 2 SD, which could indicate that the food options available in the school canteens do not adequately contribute with these nutrients. Additionally, a low level of iodine was observed, which only reached the target value with mean + 2 SDs.

According to the Nordic Nutrition Recommendations (31), target values should follow age- and sex-specific recommended intakes. As previously mentioned, we applied target values that include all sexes and age groups in our analysis (26). It is likely that most purchases made in school canteens are by individuals aged 15–19 years. When considering age and sex, the nutrient recommendations become more specific. Contributions of folate, iron (for boys) and iodine from the procurement fall within mean + 1 SD, indicating that targets may be assumed to be met throughout the day. However, vitamin D for both sexes and iron for girls emerge as key nutrients of concern.

To increase the contribution of vitamin D from procurements, one option could be to purchase fortified food alternatives. There are few fortified products for sale in Norway, aside from 0.5% fat milk that has been fortified with vitamin D. The schools in our sample primarily purchased 1% fat milk, which is not fortified. The food and meal guidelines (9) recommend low-fat milk and emphasise the use of milk with less than 0.7% fat if only a single milk option is available. Therefore, opting to procure the 0.5% fat milk could increase the contribution of vitamin D from school purchases and be in line with the guidelines.

One opportunity with public food procurement is to ensure adequate nutrition for all students, helping to reduce social inequality. The food offered in school will not provide all the necessary nutrients throughout the day; however, identifying key nutrients of concern can be invaluable in guiding meal planning and food procurement, especially in a school setting. As observed in a national dietary survey amongst 9- and 13-years olds (32), the intake of saturated fat from meat products and full-fat dairy was of concern, along with added sugar from sweets and sugar-sweetened beverages. The recent national dietary survey of adults (18–80 years) (24) indicated that high intake of saturated fat continues into adulthood. Choosing products that are both more environmentally friendly and nutritionally beneficial for students would benefit public procurement practices in upper secondary schools.

In our estimation of the environmental impacts of food procurement, we found that red meat and dairy products contributed the most between the food groups. Red meat, in particular, had a substantial environmental impact relative to its small share of the total purchases by weight. Similar findings have been reported for food procurement in two schools in Copenhagen, Denmark (19), and in school canteens in Ames, Spain (33), illustrating higher emissions per unit for animal-based foods compared to other food groups. Animal-based products are recognised as high-impact foods, thus hold potential for mitigating the environmental burdens through dietary adjustment (34). In analyses of emissions from public procurement in Norwegian counties and municipalities (20), it was estimated that emissions could vary from an increase of 2% to a decrease of 20%, depending on whether high-emission or low-emission meat sources were chosen and what type of replacements were used for meat. Based on these findings, the potential for reducing environmental impacts in upper secondary schools’ food procurement would largely depend on decreasing the share of ruminant meat. Whilst red meat accounts for only about 3.5% of total purchases, it has a relatively large environmental footprint, particularly for land use, and a reduction will disproportionally lower this footprint. In our study however, we identified a large relative impact especially of dairy products across multiple environmental impacts, due to the large share of dairy products amongst purchases. Additionally, although fish and seafood provide important nutrients, these purchases can contribute to overfishing and depletion of fish stocks both locally and globally (35), and purchases should therefore be limited to sustainably sourced fish. Therefore, a reduction in purchases of full-fat cheese, as we have advised considering the high contribution of saturated fat from procurements and an increase in plant-based foods and sustainably sourced fish, could lessen the environmental burden of school canteen purchases and be in line with the guidelines for food and meals.

Lengle et al. (36) investigated the environmental impacts of self-selected diets amongst Norwegian adults (*n* = 1,787), using the same LCA food database as employed in our study. Although their study is not directly comparable to ours, since they assessed the impact of consumed diets and we examined the impact of food purchases, it is interesting to note how the findings align. Overall, our results compared well per 10 MJ for most environmental impacts. Notably, the global warming potential of food procurement in our study was lower than that reported by Lengle et al. (36). This discrepancy can be attributed to several factors: Lengle et al. analysed the entire daily intake, which included a relatively higher proportion of meat, whereas our study focused on purchases primarily for one meal and consisted of a higher proportion of beverages, which have a lower environmental impact.

We observed significant differences in purchases of most food groups between the school canteens. It is possible to have variation in purchases without compromising adherence to the guidelines for food and meals. For instance, variation may be a result from local food procurement, which can serve as a valuable tool for supporting the local community and culture through purchasing decisions (5). However, due to the discrepancy between school purchases in our sample and the food and meal guidelines, the variability in purchases may suggest a lack of strategic tools and guidance for food procurement in upper secondary schools. Therefore, establishing monitoring mechanisms and providing strategic guidance could be essential steps to ensure procurements are both consistent and effective whilst also identifying targeted strategies to support local food culture.

Food procurement relies on the political, economic and legislative landscape, which may pose challenges in terms of different goals and priorities. For instance, although the Public Procurement Act in Norway mandates that public procurement consider environmental sustainability, this obligation can sometimes conflict with the law’s overall goal to ‘promote the efficient use of society’s resources’ (37). As argued by various stakeholders involved with public food procurement in Sweden (38), it is possible to purchase higher quality and sustainable foods by shifting from the traditional low-cost model towards incorporating higher quality criteria into the evaluation processes. Therefore, through their decision-making and budget allocations, local politicians and administrative leaders have a responsibility to ensure healthy and sustainable public food procurement.

Additionally, schools play a crucial role in shaping the food environment and can thereby influence students’ eating behaviour (39), as demonstrated in a Dutch study (17), improving the food selection in school canteens may not automatically promote healthy habits amongst the students. Nonetheless, research works have shown that the selection of foods available in school and in the nearby area can increase the risk of obesity, whereas the opposite was seen with the availability of healthy foods (40).

### Strength and limitations

A strength of this study is its access to a comprehensive overview of school food procurement data, obtained from systematically collected electronic invoice information. Although the exact volume in grams procured by the schools was estimated based on product name, quantity, packaging type and price, which introduces potential information bias, this study still provides valuable insights. By evaluating both the quantity and type of food items, along with their environmental impacts, we offer a nuanced understanding of how upper secondary schools can enhance the nutritional quality of meals and promote sustainable food purchasing.

Procurement data were obtained for either the first or second quarter of 2022, and seasonal differences may exist in the data. For instance, easter may have affected procurement and led to an increase in particular foods and beverages typical of this time of year. However, since easter occurred in mid-April in 2022 and thereby present in the second quarter procurement data, school activities may have occurred in late March, thereby affecting purchases in the first quarter procurements. Moreover, although schools were operating normally during the data collection period, the coronavirus pandemic in the preceding years may have influenced school food procurement.

We analysed purchases made by school canteens; however, the proportion of food purchases actually consumed by the students is unknown. This uncertainty affects our ability to compare the findings to the recommendations for dietary planning for groups (26) and guidelines for food and meals in upper secondary schools (9). The use of data from the Norwegian Food LCA database version 01 (22), which represents foods available on the Norwegian market, is a strength of the present study. However, the database comprises individual estimations of foods, influenced by methodological differences that introduce uncertainty in the estimation of environmental impacts. This warrants caution when comparing the environmental impacts between different foods and studies. Furthermore, our analysis did not account for avoidable food waste, which could potentially reduce the contribution of nutrients and exacerbate the environmental impact of the procurements.

The sample of schools is limited to a single county in Norway. Additionally, some of the schools participated in an intervention for more sustainable and healthy school canteens (41). Therefore, it is uncertain whether the findings from the present study can be generalised to current practices in other upper secondary schools in the county and across the rest of Norway, aside from indications of the current nutritional quality and environmental impact.

Several dimensions of sustainability, such as economic and social sustainability, were not investigated in our study. Additionally, our analysis was limited to four environmental impacts available in the Norwegian food LCA database (22), leaving certain aspects of the sustainability of food procurements in the upper secondary schools unaddressed.

Furthermore, although public food procurement could be a vital tool, it should be integrated with other policies and strategies aimed at enhancing healthy and sustainable meals in schools. These may include reducing food waste, making improvements in other phases of the product value chain, food education, and encouraging healthy and sustainable meals at home.

### Future research

The use of purchase data in research can be a powerful tool for monitoring the nutritional quality and environmental impact of public food procurement. However, as our study has shown, these data currently require extensive processing to be useful. Manually estimating volumes in grams would not be feasible for larger datasets, and reporting volumes in grams in the raw data would enhance the validity of our results. Future studies could explore ways to standardise input from suppliers to include volume in grams or investigate differences in purchasing patterns between schools. Additionally, combining purchase data with food waste data could offer a more accurate assessment of nutritional quality and environmental impact.

## Conclusion

This study revealed limitations in food procurement across upper secondary schools in a large Norwegian county, which did not comply with the guidelines for food and meal in schools. Significant variability in food procurement practices that do not meet the guidelines suggests that food procurement in school may benefit from clearer guidance for canteen staff and continuous evaluation of the nutritional quality and environmental impact of purchases.

## Supplementary Material


