# Supplementary material for: Food procurement in upper secondary schools in a Norwegian county: nutritional quality and environmental impacts
Source: Food Nutr Res. 2025 Nov 11;69:10.29219/fnr.v69.12600. doi: 10.29219/fnr.v69.12600 (PMC12664301; doi:10.29219/fnr.v69.12600)
Supplement: Supplementary file 1 [file FNR-69-12600-s1.pdf]

SUPPLEMENTARY MATERIALS

Supplementary Table 1: Procurement specifications for products and meal services associated with the dietary guidelines, copied and translated from Table 2A in the guidelines "Nutritional considerations in public procurement of foods and beverages" (5)

#	Food/ product	Basic assortment	Specifications
1	Vegetables, fruit and berries	Varied selection, for all meals, in individual dishes, and side dishes, snacks. Potatoes are included.	All varieties, fresh, frozen, conserved [a]
2	Pulses (beans, lentils, etc.)	For vegetarian dishes and in combination with fish, poultry, and other lean meats.	All varieties, fresh, frozen, conserved [a]
3	Bread and cereals	Products with a high share wholemeal, whole grains, fibre, and low content of fat, sugar, and salt. Applies to bread, bakery goods, groats, grain products, breakfast cereals, whole grain rice, pasta, and coarse bulgur, couscous, barley groats, etc.	In coarse bread, > 50% of the flour mixture is wholemeal flour, whole grains, or bran. Bread and grain products that meet the requirements of the Keyhole regulation and/or the Bread Scale labelling coarse (3/4) or extra coarse (4/4).
4	Spread for bread and other lunch meals	Varied. Always vegetables and fish, preferably fruits. Otherwise, semi-fat and low-fat cheese, vegetarian pâté, hummus, spreads made of poultry and other lean meats, liver pâté, eggs, "prim" and brown cheese.	Semi-fat (maximum 17% fat) and low-fat cheese and other spreads as mentioned. Fatty fish. Avoid sweet spreads, choose alternatives with less added sugar.
5	Edible fats	Soft margarine (table margarine) for bread and soft/liquid margarine or cooking oils in cooking and in dressings.	Products with a maximum of 20% saturated fatty acids.

6	Milk and dairy	With less fat, sugar, and salt; semi-fat and low-fat cheeses, low-fat versions of milk and fermented products. Have an overview of available products in the market.	Low-fat milk 0.5% fat[b], skimmed milk. Plain yogurt, quark/ kesam, etc., with a maximum of 4% added sugar. Cheese with a maximum of 17% fat.
7	Fish and meat	Fatty- and lean fish and fish products. Choose lean meat and lean meat products. Limit the amount of processed meat and red meat. Choose white meat, lean meat, and lean meat products with less salt.	Processed fish and meat products with at least 50% fish and meat content and low in saturated fat and salt. Vegetarian alternatives and vegetable products with at least 60% vegetable ingredients.
8	Eggs	Included in a varied diet/food offer.	Various forms, such as boiled, omelette, scrambled.
9	Dressings and sauce	Dressing made of oil/water and vinegar or low-fat dairy products (see number 6). Low-fat sauces.	Low in salt and fat, maximum 20% saturated fat.
10	Products high in fat, salt and sugar	Limit the availability of cordial, nectar, sweet biscuits and pastry, chocolate, candy, and snacks. Always offer alternatives such as fruits, vegetables, etc.	Alternatives with the lowest possible content of sugar, saturated fat, salt. Light products may be suitable for adults.
11	Beverages[c]	Water, coffee, tea. Milk, see item 6. Juice rather than nectar.	Bottled water, or from a dispenser, etc. Tea and coffee, decaffeinated alternatives.

[a] Canned produce may contain a small amount of added salt or sugar

[b] Organic low-fat milk currently has 0.7% fat and is included.

[c] Beverages with sweeteners do not provide energy, but like carbonated drinks, they can cause acid damage to teeth.

Supplementary table 2*:* Guidelines for food and meals in upper secondary schools (part 3), copied and translated from the Norwegian Directorate of Health (7)

#	**Guidelines**
1	Arrangements should be made for meals that promote the enjoyment of food, social interaction, well-being and health, through the canteen and other food and drink offers.
2	Students should be ensured enough time to eat, a minimum of 20 minutes.
3	Facilities for handwashing before the meal should be provided.
4	Storage, preparation, serving, and labelling of foods must be done in accordance with regulations and advice from the Norwegian Food Safety Authority.
5	Consideration should be given to students with food allergies and intolerances.
6	The food and drink offerings should be based on the dietary guidelines of the Norwegian Directorate of Health
7	The healthiest option should be affordable and easily accessible.
8	Vegetables and fruits/berries should be offered daily.
9	Bread and grain products with a high fibre and whole grains, and low in fat, sugar, and salt should be used.
10	The selection of spreads for bread should be varied and always include fish spreads and vegetables.
11	Hot meals should consist of a variety of fish, meat, and vegetarian dishes.
12	Vegetable oils and liquid or soft margarine should be used instead of hard margarine and butter.
13	Foods with a low salt content should be prioritized, and the use of salt in cooking and on food should be limited.
14	Cold drinking water should always be available, both as a thirst quencher and served with meals.
15	Low-fat milk with 0.7 % fat or less, skimmed milk, or/or low-fat milk should be offered daily.
16	If juice is offered, the portions should not exceed 250 ml.
17	Soft drinks, cordial, and other beverages with added sugar or sweeteners should not be offered.

18	Pastries and other products high in sugar and/or fat should be reserved for special occasions.
19	Chocolate, candy, chips, and other snacks should not be offered.
20	An environmentally friendly practice should be aimed for, with minimal food waste and a menu that emphasizes plant-based foods and fish.

Supplementary Table 3: Description of food procurement from 35 upper secondary school canteens in either Q1 or Q2 of 2022.

Food groups	Mean (SD)	Median		
	kg	kg	%[a]	Q1-Q3 (kg)
Bread, cereals and grains	708 (513)	608	17	(454-764)
Cereals and grains	307 (243)	269	48	(118-399)
Flours, whole grain	162 (195)	105	59	(12-219)
Rice	12 (18)	1	1	(0-14)
Pasta	54 (34)	45	25	(27-76)
Breakfast cereals	48 (65)	13	7	(3-79)
Tortilla, chapati	30 (42)	13	7	(2-43)
Bread and crispbread	400 (487)	287	52	(73-482)
White	206 (254)	127	67	(22-292)
Semi-coarse	19 (51)	0	0	(0-16)
Coarse	149 (303)	56	30	(8-145)
Extra coarse	24 (50)	7	4	(1-15)
Unspecified	2 (5)	0	0	(0-0)
Vegetables, potatoes, legumes	648 (402)	564	15	(401-850)
Potatoes	124 (138)	91	17	(35-147)
Legumes	33 (39)	21	4	(7-40)
Vegetables	491 (280)	438	80	(291-603)
Fruit and berries	332 (583)	166	5	(99-273)
Fresh and frozen	305 (572)	153	89	(88-258)
Jam, fruit preserves	27 (27)	18	11	(4-39)
Nuts and seeds	5 (7)	3	0	(1-8)
Dairy products	803 (387)	769	21	(549-1068)
Milk	471 (280)	455	62	(241-601)
Milk, low-fat	418 (261)	413	92	(204-584)
Milk, full fat	53 (70)	35	8	(8-71)
Yoghurt	105 (91)	78	11	(32-143)
Sugar-sweetened	61 (72)	44	64	(18-77)

No sugar/artificially sweetened	44 (54)	24	36	(10-58)
Cheese	147 (82)	130	18	(87-200)
White cheese	145 (80)	130	100	(86-197)
Fatty cheese (>20% fat)	130 (70)	124	95	(74-176)
Low-fat cheese (<20% fat)	14 (30)	7	5	(2-12)
Brown cheese	2 (5)	0	0	(0-1)
Other dairy[b]	80 (52)	74	10	(30-121)
Meat and poultry	307 (192)	251	7	(162-419)
Red meat	158 (145)	112	50	(66-196)
Whole and minced red meat	50 (51)	40	38	(15-60)
Processed red meat	108 (112)	65	62	(39-141)
Poultry	149 (114)	114	50	(77-208)
Whole and minced poultry	123 (93)	94	88	(51-181)
Processed poultry	26 (39)	13	12	(4-26)
Fish and seafood	31 (35)	21	1	(4-43)
Lean and semi-fat fish	5 (9)	0	0	(0-5)
Fatty fish	6 (13)	0	0	(0-6)
Pre-fried and/or breaded	10 (18)	0	2	(0-9)
Shellfish	4 (5)	1	36	(0-6)
Fish spread (incl. smoked, graved)	6 (8)	2	61	(0-7)
Eggs	87 (74)	63	2	(36-121)
Edible fats	72 (40)	66	2	(41-98)
Fats, plant-based	54 (35)	45	77	(35-63)
Fats, animal-based	18 (16)	14	23	(4-30)
Beverages	1034 (711)	947	26	(498-1492)
Bottled water	264 (222)	218	30	(122-325)
Coffee and tea	141 (126)	123	17	(53-182)
Juice and smoothies	113 (101)	94	13	(48-153)
Other beverages	1 (3)	0	0	(0-0)
Soft drinks, energy drinks and cordial	515 (528)	294	40	(73-898)
Sugar-sweetened[c]	323 (388)	132	73	(47-528)

Artificially sweetened[d]	193 (292)	48	27	(0-334)
Discretionary foods[e]	93 (98)	58	2	(27-117)
Meat and dairy analogues	14 (19)	3	0	(0-22)
Miscellaneous[f]	173 (108)	157	4	(75-231)
Total	4306 (2113)	4164	100	(2512-5198)

Data presented as mean (SD), median (kg), and median percentage distribution and distribution expressed as Q1 and Q3).

[a] The median percentage distribution of subgroups was calculated using the total sum of subgroups instead of the main food group to equal 100.

[b] Includes cream, sour cream etc. and dairy-based desserts.

[c] Mainly sugar-sweetened iced tea.

[d] Mainly artificially sweetened soda.

[e] Includes cakes, cookies, sweet pastry, chips, popcorn, chocolates and other sweets and snacks, sorbet, however not dairy-based desserts.

[f] Includes sauces, dressings, mayonnaise, spices and salt, powders, sugars, and other additives and ingredients.
